# Down-Regulation of miR-129-5p Inhibits Growth and Induces Apoptosis in Laryngeal Squamous Cell Carcinoma by Targeting APC

**DOI:** 10.1371/journal.pone.0077829

**Published:** 2013-10-23

**Authors:** MingHua Li, LinLi Tian, Lin Wang, HongChao Yao, JiaRui Zhang, JianGuang Lu, YaNan Sun, Xu Gao, Hui Xiao, Ming Liu

**Affiliations:** 1 Services of Head and Neck Surgery, department of Otolaryngology-Head and Neck Surgery, The Second Affiliated Hospital of Harbin Medical University, Harbin, China; 2 Services of Laryngology, department of Otolaryngology-Head and Neck Surgery, The Second Affiliated Hospital of Harbin Medical University, Harbin, China; 3 Department of Biochemistry and Molecular Biology, Harbin Medical University, Harbin, China; Barts & The London School of Medicine and Dentistry, Queen Mary University of London, United Kingdom

## Abstract

miRNAs regulate gene expression and are key mediators of tumourigenesis. miR-129 has diverse effects in tumours, but its role in laryngeal squamous cell carcinoma (LSCC) remains unknown. This article focuses on the role of miR-129-5p in LSCC. We show miR-129-5p is upregulated in primary LSCC tumours and correlated with advanced disease. Down-regulating miR-129-5p suppressed cell proliferation and migration, and caused cell cycle arrest in Hep-2 cell lines. Downregulation of miR-129-5p alone is sufficient to induce apoptosis both in vivo and in vitro. Moreover, the growth of LSCC xenograft exposed to miR-129-5p antisense oligonucleotides (ASO) in BALB/c mice was markedly inhibited. In addition, we found that miR-129-5p targeted adenomatous polyposis coli (APC) to release inhibition of Wnt signalling causing cell growth and tumourigenesis. Our results suggest miR-129-5p functions as an oncogene in LSCC by repressing APC and is a potential therapeutic target for LSCC.

## Introduction

Laryngeal cancer is a common malignant neoplasm. There are an estimated 130,000 new cases each year worldwide and more than 95% are squamous cell carcinomas [1]. Treatment options after diagnosis include surgery, chemotherapy, or radiation therapy. Patients with early-stage laryngeal cancer can be effectively treated with single or multi-modal therapy, but most patients diagnosed with advanced-stage laryngeal cancer die of recurrence and/or metastasis. The survival rate of patients with laryngeal squamous cell carcinoma (LSCC) has not improved dramatically in the past 20 years and a great deal of research has been dedicated to understanding the mechanisms of tumour invasion and metastasis. The elucidation of molecular pathways involved in carcinogenesis of LSCC will provide important insights and help direct development of improved anticancer therapies.

MicroRNAs (miRNA) are small, conserved, and non-coding RNA sequences that can down-regulate gene expression by targeting the 3′-UTR region of specific mRNA sequences leading to translational repression or degradation [2], [3]. miRNAs are involved in complex genetic pathways and are essential to cellular and organismal function [4]. Alterations in miRNA expression has been implicated in carcinogenesis and metastasis [5], [6]. Differential expression of miRNA has been widely described in solid tumours compared to normal tissue and suggests that miRNAs may function as putative oncogenes or tumour suppressor genes.

We have previously demonstrated that miR-21, an onco-microRNA in many tumours, was also upregulated in LSCC and increased expression levels were correlated with advanced stages of LSCC. miR-21 significantly elevated the expression of the oncogene Ras *in vitro*, suggesting possible oncogenic effects through activation of Ras pathways in LSCC [7]. Alternatively, miRNAs such as miR-206 have tumour suppressor function. miR-206 is down-regulated in LSCC and restoring miR-206 expression inhibited cell growth, increased apoptosis, and inhibited vascular endothelial growth factor (VEGF) expression [8]. miRNAs expression profiles are abnormal in LSCC, and it is necessary to further investigate the roles of miRNAs in the development of LSCC.

LSCC has been suggested to be a human papilloma virus (HPV)-dependent malignancy. Studies have shown that several miRNAs, including miR-129, are differentially expressed in HPV-infected cells and cell lines [9], [10]. Human miR-129-1 is one of the seven miRNAs identified near FRA7H, a fragile site in chromosome 7q32 [11]. miR-129-1 is frequently detected in solid tumours [11–17and despite that its expression is down-regulated in some types of cancers [12], [14]–[16], its expression is elevated in LSCC [18]–[20]. However, the role of miR-129 in LSCC remains unknown.

In this study, we used real-time PCR to measure the expression of miR-129-5p in both cell lines and primary LSCC samples. We found that miR-129-5p was significantly upregulated in LSCC and closely correlated with clinical pathological findings of patients. We further investigated the direct effect of miR-129-5p on proliferation, cell cycle progression, and apoptosis in LSCC cells. We also demonstrated using luciferase reporter assays, that adenomatous polyposis coli (APC) is a target for miR-129-5p. APC is a tumour suppressor gene and an important regulator of the Wnt signalling pathway. Therefore, we investigated expression of downstream target proteins in the Wnt pathway, such as c-myc and cyclin D1. Our findings suggest that miR-129-5p may stimulate oncogenesis in LSCC by targeting APC and altering the Wnt signalling pathway.

## Materials and Methods

### Ethics Statement

Laryngeal squamous cell carcinoma tissues were obtained from the Department of Otorhinolaryngology at the Second Affiliated Hospital of Harbin Medical University. Written informed consent was obtained from all patients. Animals had free access to food and water, maintained in sterile micro-isolator cages under specific-pathogen-free conditions at 27±1°C and 50%±10% humidity, and exposured to a 10-hour lights on/14-hour dark cycle. Moreover, all BALB/c nude mice were under ether anesthesia before they were sacrificed. All animal handling and experimental procedures were performed in accordance with the guidelines of the Care and Use of Laboratory Animals published by the China National Institution of Health to ensure the implementation of the animal welfare measures. This study protocol was approved by the Institution Research Board of Harbin Medical University. (Approval number:HMUIRB20130003).

### Samples

Thirty-six patients (11 females and 25 males, aged 45–74 years) with laryngeal squamous cell carcinoma (stages I–IV) who underwent partial or total laryngectomy in the Department of Otorhinolaryngology at the Second Affiliated Hospital of Harbin Medical University between 2009 and 2010 were enrolled in this study. Cancerous tissues and corresponding adjacent normal tissues were collected during surgery. Samples were immediately snap-frozen in liquid N2 for 5 min and stored at −80°C. A diagnosis of squamous cell carcinoma was confirmed by histopathology. The clinical features of all patients were analysed and shown in Table 1. No patients received any therapy prior to surgery and all patients gave their informed written consent. The research protocol was approved by the hospital's Protection of Human Subjects Committee.

**Table 1:** Relationship between miR-129-5p expression levels and clinical pathology in LSCC patients.

Characteristics	N	miR-129-5p expression(aT/N Ratio)	*P*
Sex			0.88
Male	25	4.25±3.13	
Female	11	4.09±2.54	
T classification			0.04
T1–2	23	3.43±2.82	
T3–4	13	5.57±2.69	
Lymph node metastasis			0.02
Negtive	23	3.34±2.66	
Positive	13	5.74±2.83	
Defferentiation			0.37
well	22	3.84±2.60	
moderately/poorly	14	4.77±3.39	
Clinical stage			0.01
I–II	21	3.15±2.32	
III–IV	15	5.68±3.12	

a Tumor/normal (T/N) ratio: The fold change in miR-129-5p expression in LSCC to the corresponding adjacent normal tissue. miR-129-5p expression was measured by real-time PCR and normalized to the external control (human U6 gene). Values were quantified using the 2^−ΔΔCt^ method. Values are means±SD.

### miRNA isolation and quantitative real-time PCR

miRNA was extracted from tissues and the Hep-2 laryngeal squamous carcinoma cell line using Trizol reagent (Invitrogen, Carlsbad, CA) according to the manufacturer's protocol. The cDNA was reverse transcribed using All-in-One™ miRNA Q-PCR Detection Kit (Genecopoeia, Germantown, MD) at 37°C for 60 min followed by 85°C for 5 min. Real-time PCR was performed using SYBR-Green Master Mix (ABI, Foster, CA) on a 7500 Fast Real-Time PCR system (Applied Bio-System, Foster City, CA). Reaction conditions were 95°C for 10 min followed by 40 cycles at 95°C for 10 s, 57°C for 20 s, and 72°C for 15 s. Expression data calculated from the CT value were normalized to expression of the human U6 gene in each sample using the 2^−ΔCt^ method. The primers for miR-129-5p were: forward, 5′-GATCCGCAAGCCCAGACCGCAAAAAGTTTTTA-3′ and reverse, 5′-AGCTTAAAAACTTTTTGCGGTCTGGGCTTGCG-3′. Each sample was measured in triplicate.

### Luciferase reporter assay

The human APC 3′UTR (base 404–411) was amplified and cloned into the multiple cloning sites in a psi-CHECK™-2 luciferase miRNA expression reporter vector. Site-directed mutagenesis of the miR-129-5p target site in the APC-3′-UTR was used as a negative control and termed Mut-3′-UTR. For the reporter assay, cells were transiently transfected with luciferase reporter gene constructs and miR-129-5p or its antagomir targeting endogenous miR-129-5p. Transfection was performed in 24 well plates using lipofectamine 2000 (Invitrogen, USA). Luciferase activity was measured 48 h after transfection using a dual luciferase reporter system according to the manufacturer's protocol (Promega). Firefly luciferase activity was normalized to renilla luciferase activity for each transfected well and 3 independent transfection experiments were performed in triplicate for each plasmid construct.

### Lentivirus vectors for anti-sense oligonucleotides of miR-129-5p

A recombinant lentivirus encoding an ASO against human miR-129-5p and a control lentivirus were artificially synthesized by Genechem (Shanghai, China) and tittered to 10^9^ TU/mL for preparation according to manufacturer's protocol. To monitor transfection, both recombinant lentiviruses contained a green fluorescent protein (GFP) sequence.

### Cell culture and transfection

The human LSCC cell line Hep-2 was purchased from The Cell Bank of Chinese Academy of Science (Shanghai, China). Cells were maintained in DMEM (ThermoFisher Scientific, Waltham, MA) supplemented with 10% foetal bovine serum (Shenggong, Shanghai, China)and incubated at 37°C under humidified atmosphere containing 5% CO_2_. Hep-2 cells in the logarithmic growth phase were seeded in 6-well plates at a concentration of 1×10^5^ cells per well. After 12 h, cells were at ∼40–50% confluence and were transfected by adding 1 mL of complete medium containing lentivirus (10^8^ TU/mL) and polybrene (8 mg/mL) to each well. Cells were incubated at 37°C for 12 h, followed by incubation in DMEM medium containing 10% foetal bovine serum and 1% penicillin-streptomycin for the another 24 h. Culture medium was replaced with fresh DMEM and at 72 h post-transfection, the mean percentage of GFP-positive cells in the Hep-2 cultures was calculated in 3 random fields-of-view (FOV) per well using a fluorescence microscope (IX70, Olympus, Japan) at 200× magnification.

### CCK8 cell proliferation assay

Untransfected Hep-2 cells were used as controls for cells transfected with ASO-miR-129-5p lentivirus or GFP-lentivirus. Cells were plated in 96-well plates at a density of 2×10^3^ cells per well at day 0. To measure cell proliferation, 10 µL CCK8 reagent (C0038, Beyotime Inst Biotech, China) was added to cell cultures according to the manufacturer's protocol on days 0, 1, 2, 3, and 4. After addition of CCK8, cells were incubated at 37°C for 4 h and then the absorbance at 450 nm was measured using a micro-well plate reader (Multiscan MK3; Thermo Labsystems, USA). Proliferation was measured in 5 replicate wells for each group on each day. The percentage rate of cell growth was calculated using the following formula: (mean absorbance of the treatment group/mean absorbance of the control group) × 100.

### Cell migration assay

Correlations between miR-129-5p levels and the ability of human LSCC cell lines to migrate and invade was investigated using 24-well Boyden chambers (8 mm pore size) coated with Matrigel (Becton Dickinson Labware). Seventy-two hours after transfection with lentivirus containing ASO sequences against miR-129-5p, 2×10^4^ LSSC cells were resuspended in 200 µL of serum-free medium and plated in the upper compartment of the Boyden chambers. The lower compartments contained 1 mL of medium containing 10% foetal bovine serum to serve as a chemoattractant. Cells were incubated for 24 h and then cells on the top side of the filters were removed mechanically while cells that migrated to the bottom side were fixed in 4% paraformaldehyde and stained with H&E. Migrated cells were counted by averaging the number of cells in 5 FOV at 200× magnification. Three independent experiments were performed.

### Cell cycle assay

Cells were removed from culture plates by trypsin at 72 h post-transfection and washed with PBS. Cells were fixed with 70% ice-cold ethanol for 1 h at 4°C. After washing with PBS, cells were treated with RNase A (50 µg/mL) and stained with propidium iodide (PI) (25 µg/mL) at 37°C for 30 min. Twenty thousand events for each sample were analysed using a flow cytometer (FACS Calibur; Becton Dickinson Immunocytometry Systems, San Jose, CA) and the distribution of cell-cycle phases was determined using Modfit software (LT for Mac, V 3.0).

### Apoptosis assay

Apoptosis was measured using an Annexin V-FITC and PI double-stain detection kit (Key Gen Biotech, Nanjing, China) according to the manufacturer's protocol. Briefly, cells were harvested at 72 h post-transfection and resuspended in the Annexin-binding buffer at a concentration of 1×10^6^ cells/mL. Cells were then stained with Annexin V-FITC and PI for 15 min at room temperature in the dark and immediately analysed by flow-cytometry. Untreated Hep-2 cells were used as negative controls and experiments were repeated in triplicate.

### Western blotting

Cells from the ASO-miR-129-5p lentivirus group, GFP-lentivirus group, and control Hep-2 cell group were harvested 72 h post-transfection and incubated in cell lysis buffer for 30 min on ice. Cell lysates were separated by sodium dodecyl sulphate-polyacrylamide gel electrophoresis (SDS-PAGE) (10% polyacrylamide gels) and transferred to polyvinylidene fluoride (PVDF) membranes. After blocking non-specific protein binding sites with buffer containing 5% skimmed milk in tris-buffered saline (TBS) containing 0.05% Tween20 (TBST) buffer solution, membranes were incubated with primary antibodies overnight at 4°C. Primary antibodies included a mouse anti-human cyclin D1 (1:200, Zhongshan Golden Bridge Biotechnology, Beijing, China), a rabbit anti-human APC, and a rabbit anti-human c-myc (1:200, Boster, Wuhan, China). Membranes were washed with TBST and incubated with species-appropriate HRP-conjugated secondary antibodies for 1 h at 37°C. β-actin served as a loading control on the same membrane. Bands were quantified using Image J software (NIH, Bethesda, MD).

### Animal experiments

Male BALB/c nude mice (∼20 g, 5 weeks old) were divided equally into 2 groups (8 mice for the tumour group and 8 for controls). All mice were given subcutaneous injections of 1×10^6^ Hep-2 cells suspended in 100 µL DMEM containing 10% foetal bovine serum into the dorsal scapula region. Tumour growth was measured twice per week and calculated by the following formula: 1/2×length×width^2^. After tumours reached ∼0.5–0.6 cm^3^, mice were treated once per week with lentivirus (100 µL injected into tumours). Mice in the experimental group received an injection of ASO-miR-129-5p lentivirus while control mice received GFP-lentivirus. Three weeks after the treatment, mice were sacrificed and tumours were dissected for further analysis.

### Transmission electron microscope examination (TEM)

Tumours were cut into 1 mm^3^ sections and fixed in 3% glutaraldehyde for 24 h at 4°C and 1% osmium tetroxide for 2 h. Samples were dehydrated through a gradient of ethanol and immersed in Epon 821 at 60°C for 72 h. Sections were cut at 70 nm thickness and stained with uranyl acetate and lead citrate. Sections were then observed by transmission electron microscopy (H-600, HITACHI, Japan).

### TUNEL assay

Apoptotic cells in tumour sections were detected by terminal deoxynucleotidyl transferase dUTP nick end labelling (TUNEL) using the *In Situ* Cell Death Detection Kit (R&D, USA). After routine deparaffinization, sections were digested with a proteinase K solution for 25 min, followed by a blocking solution for 15 min. Sections were then incubated with 50 µL TUNEL reaction mixture for 60 min followed by an incubation with an alkaline phosphatase antibody for 20 min. Diaminobenzidine (DAB) was used as a chromogen to enhance positive signals and slices were then counterstained with haematoxylin. After staining, sections were dehydrated and mounted. All incubations were performed at 37°C under a humidified atmosphere. A negative control was prepared by treating the samples without Terminal Deoxynucleotidyl Transferase (TdT). For quantitative analysis, the percentage of TUNEL-positive cells per 200 tumour cells were averaged from 10 randomly-selected fields of view (FOV) per section using light microscopy at 400× magnification (Olympus, Tokyo, Japan).

### Immunohistochemistry

Formalin-fixed, paraffin-embedded samples were cut sequentially into 4-µm thick slices. After deparaffinization and rehydration, slices were treated with 0.3% H_2_O_2_ to quench endogenous peroxidase activity and blocked with 10% normal goat serum for 20 minutes. Antigen retrieval was performed with Ethylene Diamine Tetraacetic Acid (EDTA) (pH 8.0) at 100°C for 20 min. Each section was incubated with a primary antibody overnight at 4°C. After incubating at 37°C for 45 min, sections were incubated with second antibodies 1 h at room temperature. Peroxidase signal was developed by diaminobenzidine tetrachloride for 10 min and sections were counterstained with haematoxylin. Negative controls were sections incubated without primary antibody (PBS only).

### Statistical analysis

Statistical analysis was performed on SPSS (version 13.0). All values are expressed as the mean ± SD. Paired Student's *t* tests were used to determine the statistical significance for pairwise comparisons. Data of cell proliferation and the growth rate of LSCC xenografts were analysed using 2-tailed *t* tests. An SNK-q test was used for comparisons from the luciferase reporter assays, real-time PCR, cell cycle assays, invasion/migration assays, TUNEL assays, and western blots. *P* values <0.05 were considered significant.

## Results

### miR-129-5p is overexpressed in human LSCC

Levels of miR-129-5p in LSCC samples from the 36 patients enrolled in the study were ∼4 fold greater compared to adjacent healthy tissue from the same patients (*P*<0.05). We also analysed relationships between miR-129-5p expression levels in the LSCC tumours and the clinical data from those patients (Table 1). There were no differences between genders regarding miR-129-5p expression, but higher miR-129-5p expression in LSCC tumours was positively correlated with T grade, lymph node metastasis, and clinical staging. Patients with higher miR-129-5p expression had higher-grade tumours, lymph node metastases, or more advanced clinical disease.

### ASO-miR-129-5p down-regulates miR-129-5p in Hep-2 cells

**Figure 1:**
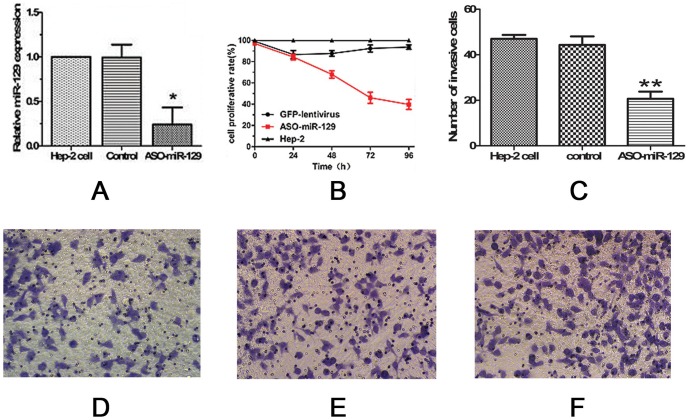
ASO-miR-129-5p down-regulates miR-129-5p and reduces proliferation and migration of Hep-2 cells . (A) miR-129-5p expression was significantly decreased after ASO-miR-129-5p transfection compared to GFP transfection (control) or untreated Hep-2 cells (Hep-2 cell) measured by real-time RT-PCR. (* *P*<0.05). (B) Less proliferation was measured in the ASO-miR-129-5p group compared to control groups at 48, 72, and 96 h post-transfection (*P*<0.05). (C) Average number of Hep-2 cells in each group that migrated to the lower chambers of transwell plates in FOV (**P<0.01). (D) Representative images of migrating cells in the ASO-miR-129-5p group 72 h after transfection. (E) Representative images of migrating cells in the GFP group 72 h after transfection. (F): Representative images of migrating cells in the untreated group 72 h after transfection.

The efficiency of lentiviral vector transfection was measured. The percentage of Hep-2 cells expressing GFP 72 h after lentivirus transfection was >80% (Fig. S1 A-D). Hep-2 cells transfected with the ASO-miR-129-5p lentivirus had considerably lower levels of miR-129-5p expression compared to cells transfected with the GFP-lentivirus as measured by quantitative real time-PCR. The PCR results confirmed that the ASO-miR-129-5p construct effectively suppressed miR-129-5p expression in Hep-2 cells (*P*<0.05; Fig. 1A).

### Down-regulation of miR-129-5p reduces Hep-2 cell proliferation in vitro

Proliferation of Hep-2 cells was measured after transfection with either ASO-miR-129-5p- encoding lentivirus or GFP-encoding lentivirus. At 48, 72, and 96 h post-transfection, cells transfected with the ASO-miR-129-5p virus showed significantly less proliferation than control cells (GFP transfected cells) (*P*<0.05; Fig. 1B).

### Down-regulation of miR-129-5p reduces motility of Hep-2 cells in vitro

Hep-2 cells were transfected with ASO-miR-129-5p, GFP, or no vector. At 72 h post-transfection, cell movement to the lower well of a Boyden chamber was measured. Cells transfected with ASO-miR-129-5p migrated in significantly fewer numbers (20.67±5.51) than cells transfected with GFP (44.33±6.51) or untransfected cells (47±3.61) (**P<0.01). Overall, transfection with ASO-miR-129-5p decreased cell migration by ∼65% compared to control cells (Fig. 1C).

### Down-regulation of miR-129-5p affected the progression of cell cycle in Hep-2 cells

**Figure 2:**
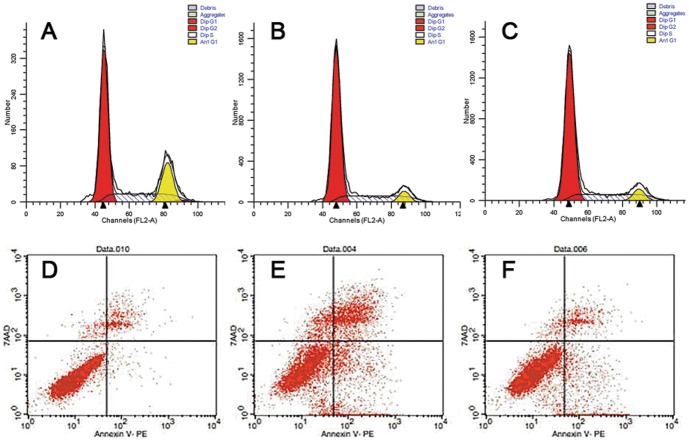
Flow cytometric analyses of the effect of ASO-miR-129-5p on Hep-2 cells' cell cycle and apoptosis. (A) Cell cycle profile of the untreated group; G0/G1 = (70.39±1.34%), G2/M = (1.53±0.53%), S = (28.09±1.33%). (B) Cell cycle profile of the ASO-miR-129-5p group; G0/G1 = (77.31±2.31%), G2/M = (1.73±1.05%), S = (20.97±1.53%). (C) Cell cycle profile of the GFP control group; G0/G1 = (71.03±1.21%), G2/M = (1.54±0.82%), S = (27.44±0.98%). One representative experiment of the 6 independent experiments is shown. (D) The percentage of apoptotic cells in the untreated group (1.87±1.44%). (E) The percentage of apoptotic cells in the ASO-miR-129-5p group (13.61±1.12%). (F) The percentage of apoptotic cells in the GFP control group (5.98±1.23%). One representative experiment of the 3 independent experiments is shown.

Transfecting Hep-2 cells with the GFP-lentivirus caused no significant changes to the cell cycle progression at 72 h post-transfection compared to untransfected Hep-2 cells (P>0.05). Transfection with ASO-miR-129-5p, however, caused the percentage of the Hep-2 cells remaining in the G1 phase increased by 7% compared to the GFP-transfected cells and by 6% compared to the untransfected cells (P<0.05). Moreover, the percentage of Hep-2 cells transfected with ASO-miR-129-5p remaining in S phase decreased by 7% compared to the untransfected cells and by 6% compared to the GFP-transfected cells (P<0.05) (Fig 2 A-C). Down-regulation of miR-129-5p significantly affected the progression of cell cycle in Hep-2 cells.

### Down-regulation of miR-129-5p enhances apoptosis of LSCC

**Figure 3:**
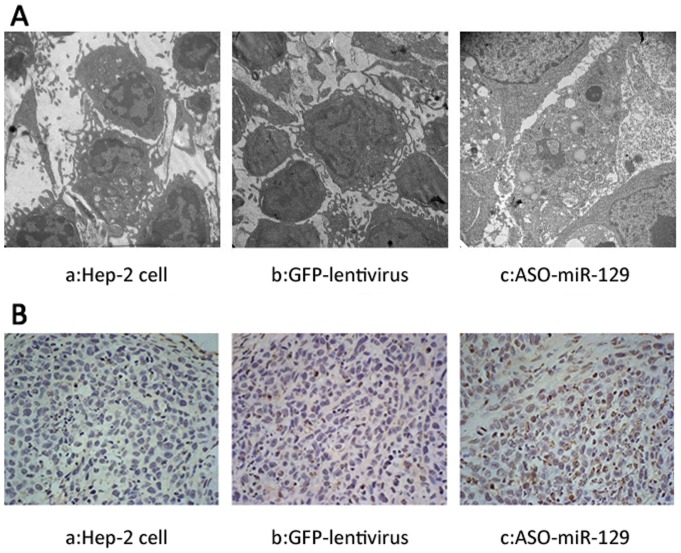
Ultrastructure of tumour cells by transmission electronic microscopy and detection of apoptosis using TUNEL assay. (A) Tumour cells exhibited the characteristic morphology of apoptosis: chromatin margination, condensation of the nucleus, and apoptotic bodies (c) after treatment of ASO-miR-129-5p, but not tumour cells treated with GFP (b) or untreated Hep-2 cells (a) (original magnification: 12000×). (B): The percentage of apoptotic cells in the ASO-miR-129-5p group (c) was significantly higher (39.01±2.32%) than in untreated group (a) (4.96±3.17%) or GFP control group (b) (6.4±1.81%). (*P*<0.05, original magnification: 400×)

Figure 2 shows the percentage of apoptotic cells in the ASO-miR-129-5p -treated group is significantly higher (13.61±1.12%) than in the untreated cell group (1.87±1.44%) or GFP-treated cell group (5.98±1.23%) (P<0.05) in cultured cells. Furthermore, in the xenograft sections from the nude mice, morphological findings from transmission electron microscopy show typical signs of apoptosis such as nuclear condensation and fragmentation, marginalization of chromatin, cell shrinkage, and formation of cytoplasmic vacuoles in tumours treated with ASO-miR129-5p lentivirus (Fig. 3 A-c). No obvious apoptosis was observed in the tumours from the GFP-lentivirus group or the untreated group. Rather, these tumour cells appeared with characteristics of healthy, dividing cells such as normal size and shape. Cells had large nuclei with prominent nucleoli and finely-dispersed chromatin (Fig. 3 A-a,b). The TUNEL assay revealed significantly greater numbers of apoptotic cells in the ASO-miR-129-5p group (39.01±2.32%) compared to the GFP-transfected control group (6.4±1.81%) or the untreated control group (4.96±3.17%) (Fig. 3B; P<0.05). These data together strongly suggest that down-regulation of miR-129-5p induces apoptosis in LSCC cells.

### Down-regulation of miR-129-5p inhibits the growth of LSCC xenografts in nude mice

**Figure 4:**
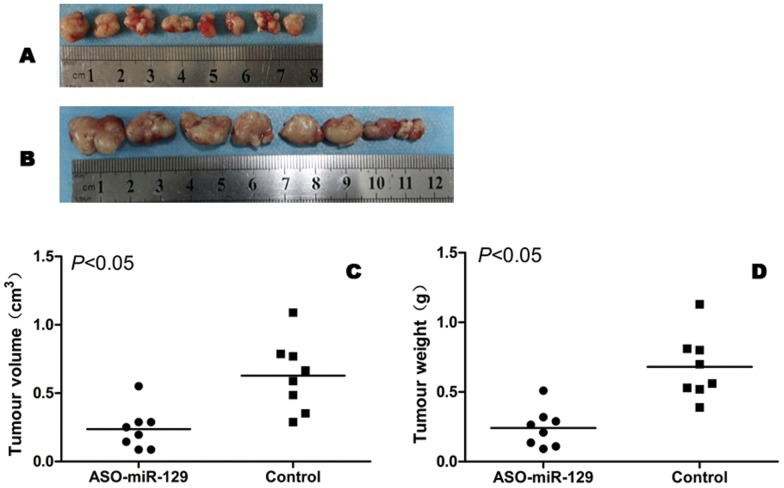
ASO-miR-129-5p inhibits the growth of LSCC tumours *in vivo*. (A) Tumours in ASO-miR-129-5p group. (B) Tumours in the GFP group. (C) The difference in tumour volumes between the ASO-miR-129-5p group and control (*P*<0.05). (D) The difference in tumour weights between the ASO-miR-129-5p group and control (*P*<0.05).

Hep-2 xenografts were implanted in 2 groups of mice. Mice were then treated with the lentiviral vectors encoding either ASO-miR-129-5p or GFP. While mice in both groups formed detectable tumours by the end of the study, the average tumour volume (0.24±0.15 cm^3^) and weight (0.24±0.14 g) in the ASO-miR-129-5p treated group was significantly lower than the tumour volume (0.63±0.25 cm^3^) and weight (0.68±0.23 g) from mice in the control group (*P*<0.05; Fig. 4). This data suggests that ASO-miR-129-5p can significantly inhibit the growth and progression of LSCC tumours *in vivo*.

### miR-129-5p is a negative regulator of APC

**Figure 5:**
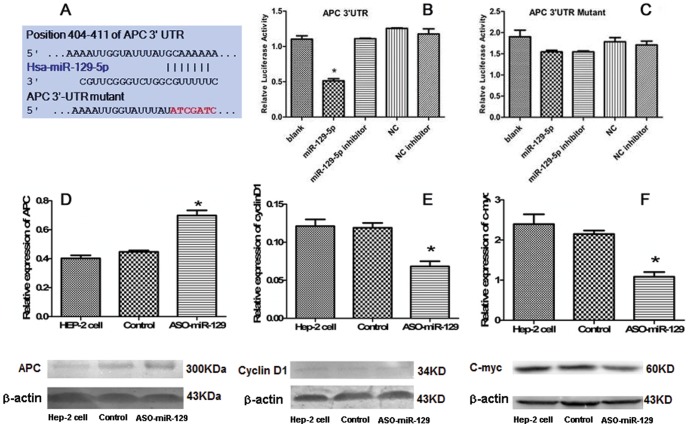
miR-129-5p directly targets APC-mRNA at 3′-UTR and protein expression of genes regulated by miR-129-5p. (A) The predicted miR-129-5p target site on the APC 3′-UTR. (B) Luciferase reporter gene assay measuring interactions between miR-129-5p and its binding site on the 3′UTR of APC in HEK293T cells. The expression of the APC reporter was significantly decreased 53% in miR-129-5p-transfected cells compared to control cells. *P<0.05. (C) Luciferase reporter gene assay for measuring interactions between miR-129-5p and 3′UTR of the APC mutant in HEK293T cells. There was no significant difference between groups (*P*>0.05). Luciferase experiments were repeated 3 times. (D) APC expression in the cells treated with ASO-miR-129-5p was increased compared to that in the controls. *P<0.05. (E) cyclin D1 expression in the cells treated with ASO-miR-129-5p was lower compared to controls. *P<0.05. (F) c-myc expression in the cells treated with ASO-miR-129-5p was lower compared to controls. *P<0.05.

**Figure 6:**
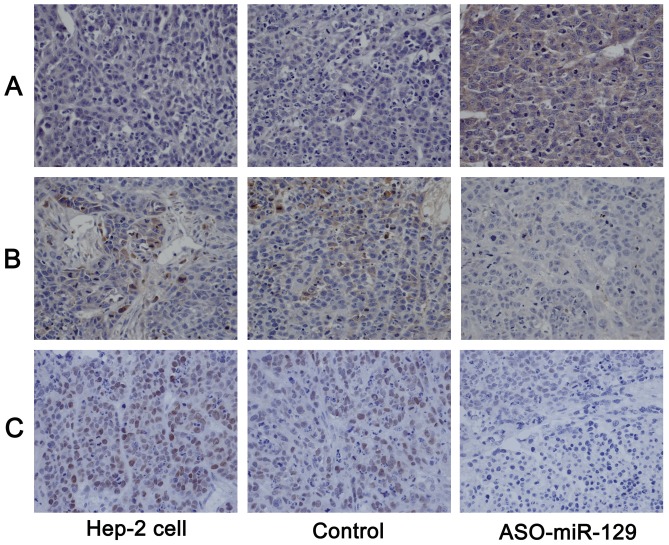
Immunohistochemistry of APC, cyclin D1, and c-myc. (A) Intense cytoplasmic labelling of APC was observed in cells treated with ASO-miR-129-5p compared with that in GFP or untreated Hep-2 cell groups. (B) Immunohistochemical signals of cytoplasmic cyclin D1 were undetectable in the sections from the ASO-miR-129-5p group, while signal is robust in sections from the GFP and untreated groups. (C) Immunohistochemical signals of cytoplasmic c-myc were undetectable in the sections from the ASO-miR-129-5p group, while signal is robust in sections from the GFP and untreated groups.

To investigate the interactions between miR-129-5p and APC, we cloned the miR-129-5p binding sites from the 3′-UTR of APC into a luciferase reporter plasmid containing a constitutively-active promoter and subsequently transfected HEK293T cells (Fig. 5A). Co-transfection of miR-129-5p with the luciferase reporter plasmid resulted in less luciferase activitiy than transfection of the reporter plasmid alone. Additionally, miR-129-5p transfection did not reduce the luciferase activity of the reporter construct transfected with mutant 3′UTR of APC. Co-transfection of the miR-129-5p inhibitor, ASO-miR-129-5p, with miR-129-5p also eliminated the ability of miR-129-5p to silence luciferase activity. Moreover, negative control (NC) miRNA did not affect luciferase activity of reporters containing either the 3′UTR of APC or the mutant APC construct (Fig.5 B, C). These results indicated that miR-129-5p directly interacts with APC. Moreover, we used western blotting and immunohistochemical staining to measure expression of APC and downstream signalling molecules, C-myc and cyclin D1 *in* vitro and *in vivo*. Down-regulation of miR-129-5p in Hep-2 cells transfected with the ASO-miR129-5p lentivirus led to higher levels of APC expression compared to cells transfected with the GFP-lentivirus or untransfected cells (*P*<0.05) (Fig. 5 D–F). Increased APC expression also correlated with lower levels of cyclin D1 and c-myc. Cells transfected with the GFP-lentivirus did not show any significant changes in expression of any of these proteins compared to the untransfected cells group (*P*>0.05). Immunohistochemical staining showed greater levels of cytoplasmic APC in cells treated with the ASO-miR129-5p-lentivirus while cells in GFP-lentivirus group or untransfected group showed only weak expression (Fig. 6A). Additionally, cytoplasmic cyclin D1 and c-myc were less prominent in the tumour tissue from the ASO-miR129-5p-treated cells group than in tumours from the GFP-lentivirus group or untreated group (Fig. 6 B and C).

## Discussion

We demonstrated in this study that miR-129-5p expression was significantly upregulated in LSCC tumour specimens compared to adjacent non-cancerous tissues in human patients. Patients with higher expression of miR-129-5p also had advanced clinical-stage disease, T3-T4 grades, and lymph node metastases. miR-129 expression differs greatly in different tumour types. For example, miR-129 expression is high in human oesophageal squamous cell carcinomas (ESCC) and retinoblastomas [11], [12], but is down-regulated in human bladder tumours, gastric cancers, paediatric brain tumours, and hepatocellular carcinomas [13], [14]–[16].

While differences in tumour types, tissue-specific differences exist [17], diverse molecular pathways in miRNA signalling and regulation are also important factors to consider when approaching the discrepancies in mRNA expression in cancer. Therefore, it is necessary to profile the molecular function and the underlying mechanisms of cancer-related miRNAs in each tumour type. Few studies have investigated miR-129 in cancers and no previous study specifically investigated the role miR-129 in cell proliferation, migration and apoptosis in LSCC. We demonstrate that down-regulation of miR-129-5p in Hep-2 cells by transfecting ASO specific to miR-129-5p significantly suppressed cell proliferation and migration. In addition, we observed that down-regulation of miR-129-5p in Hep-2 cells caused the increased number of G0/G1-phase cells and the reduced number of S-phase cells as compared to in the control cells, implying the suppression effect of cell proliferation induced by transfection of ASO-miR-129-5p in Hep-2 cells. Furthermore,tumour growth in mice was significantly inhibited by injections of the ASO-miR-129-5p lentivirus, and apoptosis of tumour cells was induced by the ASO-miR-129-5p lentivirus both *in vitro* and *in vivo*. These findings suggest that miR-129-5p may be a potential therapeutic target for LSCC.

Dual functions for miR-129, as a tumour suppressor and oncogene have been observed in different cancers. The evidence supporting its anti-tumour effects stem from observations that it was cytotoxic in both TPC-1 thyroid cancer cells and bladder carcinoma cell lines. It has been reported that miR-129 was methylated in more than 95% of esophageal squamous cell carcinoma (ESCC) [18]. Down-regulation of miR-129 through methylation was correlated with upregulation of the SRY-related high-mobility group box 4 (SOX4) in gastric cancers [14]. Restoration of miR-129 in cancer cells by pharmacological induction of histone acetylation and DNA demethylation resulted in decreased SOX4 expression [14], [18]–[20]. Histone acetylation is intimately related to miRNA expression and consequently histone deacetylase inhibitors (HDACi) such as 4-phenylbutyric acid (PBA) could alter miR-129 expression. Treating a breast cancer cell line with a pro-apoptotic dose of HDACi caused upregulation of miR-129 [20]. These findings suggest that miR-129 functions as a tumour suppressor in some cancers. In contrast however, miR-129 is also upregulated in several solid tumours and non-cancerous tissues from cancer patients with lymph node metastases [11], [12], [21]. Our results also suggest an oncogenic function of miR-129-5p, potentially through differential regulation of signal transduction pathways. miRNAs degrade or repress translation of target mRNA [4]. The functional balance between oncogenes and tumour suppressors ultimately dictates cancer development and progression. Therefore, to validate the oncogenic function of miR-129-5p, a tumour suppressor target needed to be identified. We have predicted several potential target candidates for miR-129-5p. Some of them have been discussed as oncogenes in other tissues or cell lines. Elevated expression of Valosin containing protein (VCP)/p97 in hepatocellular carcinoma (HCC) is correlated with increased incidence of recurrence. Liu et al demonstrated that down-regulation of VCP by microRNA-129-5p could suppress the genesis and progression of hepatocellularcarcinoma [16]. Epigenetic repression of microRNA-129-2 led to overexpression of SOX4 oncogene in endometrial cancer [19]. Wu et al showed that cyclin-dependent kinase 6 (Cdk6), a kinase involved in G1-S transition, is a direct target of miR-129 and downregulation of Cdk6 by miR-129 plays an important role in regulating cell proliferation in lung epithelial derived cells [22]. In consideration of negative regulation of miRNA, we notice APC, a tumour suppressor, among these potential targets for miR-129-5p.

APC is a negative regulator of the Wnt signalling pathway. Wnt is involved in both normal development and tumourigenesis. It controls multiple aspects of development such as cell proliferation, migration, polarity, and differentiation [23]–[25]. Increased Wnt signalling has been observed in several human cancers, including those of intestines [26], liver [27], colon [28], breast [29], prostate [30], kidney [31], stomach [32and oral cavity [33]. APC and Axin proteins form a complex with beta-catenin and act together with glycogen synthase kinase-3β (GSK-3β) to promote beta-catenin phosphorylation and cytoplasmic degradation. Normal levels of APC negatively regulate beta-catenin to keep it at low levels. Cytoplasmic beta-catenin interacts with a family of transmembrane cadherin (Calcium-dependent adhesion) proteins and forms complexes with alpha-catenin linking to microtubules and the actin cytoskeleton. As a result, the Wnt signalling is critical for intercellular adhesion and plays an important role in tumour invasion and growth. We identified APC as a direct target gene of miR-129-5p using a luciferase reporter assay and showed that down-regulation of miR-129-5p induced greater APC expression both *in vitro* and *in vivo*. We demonstrated that miR-129-5p regulates APC by inhibiting its expression. This likely explains the suppressed proliferation and migration capabilities of Hep-2 cells following miR-129-5p down-regulation by ASO. The role of miR-129-5p to enhance tumour growth and progression was further demonstrated *in vivo* by treating LSCC tumour-bearing mice with miR-129-5p ASO and demonstrating slower tumour growth.

Increased miR-129 leads to decreased APC expression that could cause accumulation of beta-catenin in the cytoplasm. Accumulation of beta-catenin in the cytoplasm leads to its translocation to the nucleus where it binds to T-cell factor (Tcf)/lymphoid enhancer factor (LEF) family members and activates transcription of cell growth factors like c-myc, cyclin D1, and other genes. Several studies suggest that the transcription factor c-myc is involved in aggressive cancers. It stimulates cell proliferation and regulates apoptosis and invasion. Elevated c-myc activity is a hallmark for human tumourigenesis [34], [35]. In this study, we showed that down-regulation of miR-129-5p lowered levels of c-myc both *in vivo* and *in vitro* suggesting that miR-129-5p could be targeted to regulate c-myc indirectly. Furthermore, lower levels of c-myc after down-regulating miR-129-5p could explain the increased apoptosis observed in LSCC after transfection with miR-129-5p-specific ASO.

Lastly, our results suggested that miR-129-5p may regulate cell proliferation and division by modulating cell cycle progression. Cells transition from the G1 phase into the S phase and then complete the cell division. Cyclin D1 is an important promoter of the G1-S transition during the cell cycle [36]. We showed that down-regulation of miR-129-5p by ASO-miR-129-5p transfection lowered cyclin D1 expression in LSCC both *in vivo* and *in vitro*. Accordingly, greater numbers of Hep-2 cells were observed to remain in the G1 phase after down-regulation of miR-129-5p expression by ASO.

In conclusion, miR-129-5p expression was upregulated in human LSCC. Down-regulation of miR-129-5p suppressed both proliferation and migration of tumour cells while down-regulation also increased apoptosis of tumour cells. Together, these data suggest that miR-129-5p has an oncogenic role in LSCC and that it directly inhibits the tumour suppressor APC and allows increased Wnt signalling to occur. Through increased Wnt signalling, other oncogenic factor such as c-myc and cyclin D1 are activated and drive cells toward a tumour phenotype. This study implicates miR-129-5p as a potentially valuable target for novel diagnoses and treatments in LSCC.

## Supporting Information

Figure S1 Hep-2 cells 72 h after transfection. (A) Fluorescence microscopic images of cells in after ASO-miR-129-5p transfection. (B) Light microscopic images of cells after ASO-miR-129-5p transfection. (C) Fluorescence microscopic images of cells in the GFP transfection control group. (D) Light microscopic images of cells in the GFP transfection control group.
(TIF)
