# Supplementary figures and images for: Down-Regulation of miR-129-5p Inhibits Growth and Induces Apoptosis in Laryngeal Squamous Cell Carcinoma by Targeting APC
Source: PLoS One. 2013 Oct 23;8(10):e77829. doi: 10.1371/journal.pone.0077829 (PMC3806837; doi:10.1371/journal.pone.0077829)

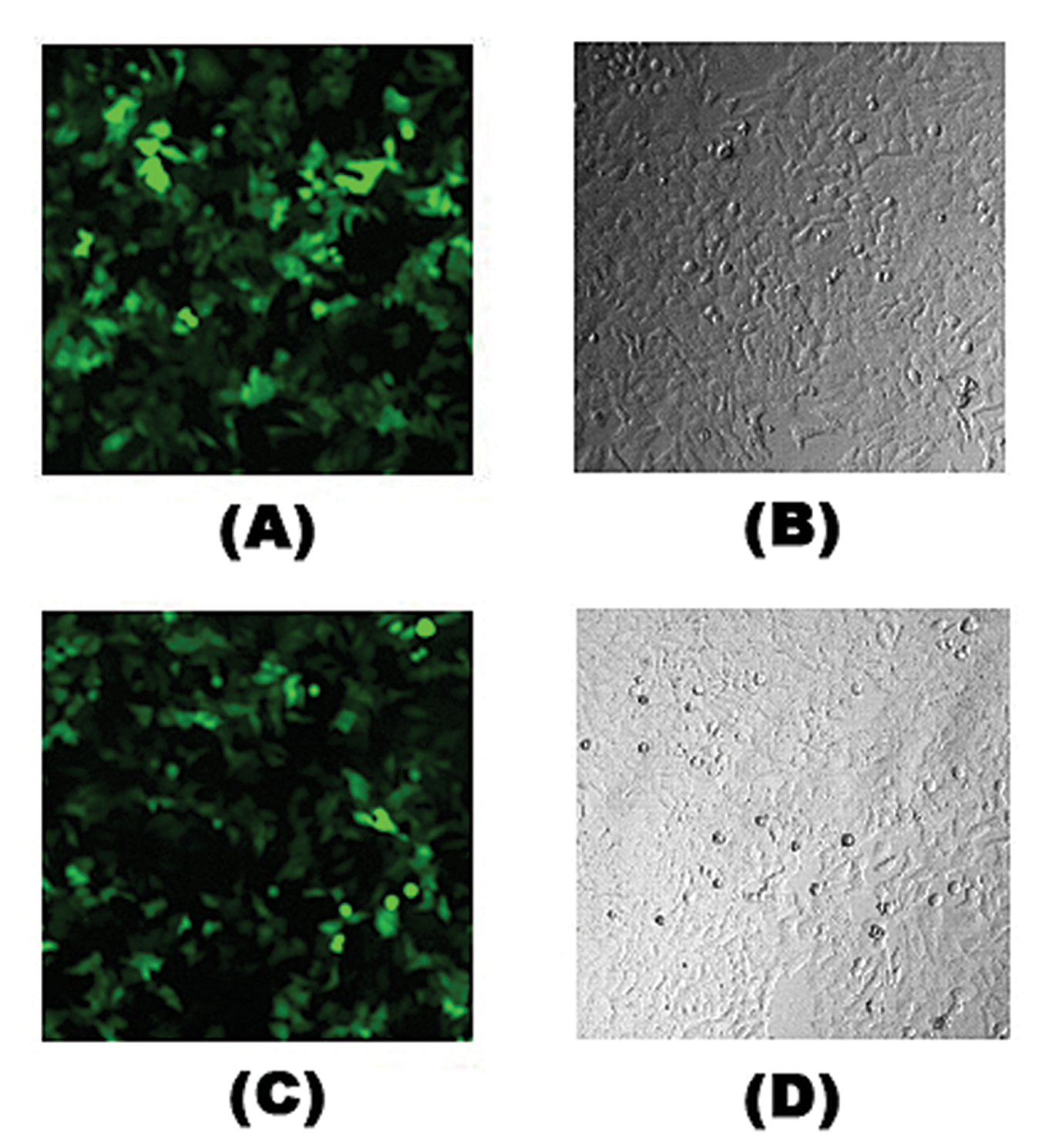

Supplement: Figure S1 — Hep-2 cells 72 h after transfection. (A) Fluorescence microscopic images of cells in after ASO-miR-129-5p transfection. (B) Light microscopic images of cells after ASO-miR-129-5p transfection. (C) Fluorescence microscopic images of cells in the GFP transfection control group. (D) Light microscopic images of cells in the GFP transfection control group. (TIF) [file pone.0077829.s001.tif]
